# Methods of measuring protein disulfide isomerase activity: a critical overview

**DOI:** 10.3389/fchem.2014.00073

**Published:** 2014-09-03

**Authors:** Monica M. Watanabe, Francisco R. M. Laurindo, Denise C. Fernandes

**Affiliations:** Vascular Biology Laboratory, Heart Institute (InCor), University of São Paulo School of MedicineSão Paulo, Brazil

**Keywords:** protein disulfide isomerase, chaperone, thiols, reduction, isomerization, oxidation, redox signaling

## Abstract

Protein disulfide isomerase is an essential redox chaperone from the endoplasmic reticulum (ER) and is responsible for correct disulfide bond formation in nascent proteins. PDI is also found in other cellular locations in the cell, particularly the cell surface. Overall, PDI contributes to ER and global cell redox homeostasis and signaling. The knowledge about PDI structure and function progressed substantially based on *in vitro* studies using recombinant PDI and chimeric proteins. In these experimental scenarios, PDI reductase and chaperone activities are readily approachable. In contrast, assays to measure PDI isomerase activity, the hallmark of PDI family, are more complex. Assessment of PDI roles in cells and tissues mainly relies on gain- or loss-of-function studies. However, there is limited information regarding correlation of experimental readouts with the distinct types of PDI activities. In this mini-review, we evaluate the main methods described for measuring the different kinds of PDI activity: thiol reductase, thiol oxidase, thiol isomerase and chaperone. We emphasize the need to use appropriate controls and the role of critical interferents (e.g., detergent, presence of reducing agents). We also discuss the translation of results from *in vitro* studies with purified recombinant PDI to cellular and tissue samples, with critical comments on the interpretation of results.

Protein disulfide isomerase is present in most eukaryotic organisms and is an essential redox chaperone that catalyzes the introduction of disulfide bonds and the rearrangement of incorrect ones in nascent proteins into the endoplasmic reticulum lumen. PDI was the first protein with folding activity described in the literature (Goldberger et al., 1964), and is the founder of the mammalian PDI family. Currently there are 20 or more other PDI members containing one or more thioredoxin-like domains (Lu and Holmgren, 2014). PDI is formed by four thioredoxin-like structural domains, two of them containing the CGHC (cysteine-glycine-histidine-cysteine) catalytic motif capable of catalyzing sulfydryl oxidation and reduction/isomerization of disulfide bonds (for a revision see Hatahet and Ruddock, 2009). The two other domains form a central hydrophobic core pocket involved in substrate binding. A mobile arm close to the C-terminal site regulates, on a redox-dependent way, substrate access to the hydrophobic core (Wang et al., 2013). In addition to its role in disulfide introduction in nascent proteins, PDI has a chaperone effect, preventing protein aggregation in a way not directly related to its redox thiols, but rather to the hydrophobic pocket region away from the redox domains. PDI binds *in vitro* to a variety of molecules, from small peptides to proteins, while *in vivo* only a few client proteins were identified (Hatahet and Ruddock, 2009). In plasma membrane and pericellular compartments, PDI is involved in important biological processes such as thrombus formation, tissue factor regulation, platelet aggregation, cell adhesion and virus internalization. The multiple PDI cellular redox effects and its versatility in binding to several proteins implicate that PDI may act as an emerging redox cell signaling adaptor (Laurindo et al., 2012) and a promising therapeutic target of several diseases (Xu et al., 2014).

There are several assays to measure PDI activity. Some assays are more specific to one particular PDI activity (e.g., thiol reduction or oxidation), while others focus in the measurement of PDI isomerase activity. Methods for PDI activity in general are used for 3 main purposes: (a) the study of protein folding by PDI to identify substrate intermediates, which requires elaborated analysis and detection by mass spectroscopy, (b) screening of PDI substrates or inhibitors, which demands fast and low-cost assays to be preferentially adopted for high-throughput system (HTPS) platforms, (c) understanding PDI function in (patho)physiological contexts by comparison of PDI activities in different experimental conditions in biological samples. Proteins such as bovine pancreatic tripsin inhibitor (BPTI) and ribonuclease T1 (RNaseT1) fit the requirements for studies of PDI-mediated protein folding, while insulin has been chosen for HTPS automation. However, PDI assays in biological samples are a considerable challenge. Some substrates commonly used for purified PDI assays (insulin or fluorescent GSSG) were already used in cell homogenates, but the interpretation is still difficult due to intrinsic interferents such as the presence of other reductants in the assay. The purpose of this review is to critically discuss the most used methods of measuring the different types of PDI activities (e.g., isomerase, oxidative refolding, reductase, and chaperone), with emphasis given to PDI in biological samples.

## An overview of PDI activity assays

Depending on the starting material, i.e., the “substrate” of PDI, one PDI activity will be preferentially measured over others. Therefore, PDI assays can be classified based on the initial redox state of the substrate. When the substrate of a protein contains scrambled disulfides and PDI catalyzes its conversion to native state (and thus the recovery of substrate activity), this assay is named “isomerase assay” (e.g., scrambled RNase isomerization). In the case of a totally reduced protein, PDI will promote “oxidative refolding” in a series of thiol oxidation/reduction cycles and possibly isomerization reactions to promote substrate gain-of-function (e.g., reduced RNase oxidative folding). PDI reductase activity assays are easier to perform and constitute the most popular in the literature. According to substrate, reductase activity is followed through increase in turbidity or fluorescence changes (e.g., insulin reduction). Finally, using proteins that do not contain disulfide bonds as substrates, PDI chaperone activity can be measured by recovery of substrate activity and/or changes in substrate protein aggregation (e.g., GAPDH aggregation).

When developing a PDI assay, it is important to keep in mind that PDI does not have a known preferential group of substrates (such as Erp57, that preferentially folds glycosylated proteins, Jessop et al., 2007) and substrates used in PDI assays were not so far proven to be physiological PDI substrates. Also, PDI concentration at ER lumen is estimated around 0.2–0.5 mM (Lyles and Gilbert, 1991), so PDI would be in excess over many substrates, a condition that is not generally mimicked in these assays. Many *in vivo* conditions are not considered in such PDI reductase activity assays: PDI cellular compartmentalization, molecular crowding inside cells (which affects protein folding stability, Zhou, 2013), and PDI recycling after substrate folding—promoted by PDI partners (e.g., endoplasmic reticulum oxidase-Ero1, Rancy and Thorpe, 2008 or oxidized peroxiredoxin-Prx4, Zito et al., 2010). Finally, another important issue is that although *in vitro* PDI chaperone and isomerase activities can be measured separately, *in vivo* PDI redox folding will not discriminate between both activities and, contrarily, seems to require isomerase and chaperone activities acting together (Laurindo et al., 2012). Thus, results obtained from *in vitro* assays should also be interpreted taking into account their limitations due to a reductionist design. A way to partially overcome such limitations is to preferentially combine the measurement of PDI activity with *in vivo* data and discuss them in the context of the specific physiological milieu being investigated.

### Isomerase assay

Assays for isomerase activity are based on gain-of-function of an inactive protein substrate containing unfolded disulfides (or scrambled disulfide bonds), so it is inactive. In the presence of PDI, disulfides bonds will be folded back to a “correct” position, thus allowing the measurement of activity of the recovered substrate. Two enzymes are more commonly used, scrambled RNase (scRNase, 4 disulfides) and riboflavin-binding protein (RfBP, 9 disulfides).

**Figure 1:**
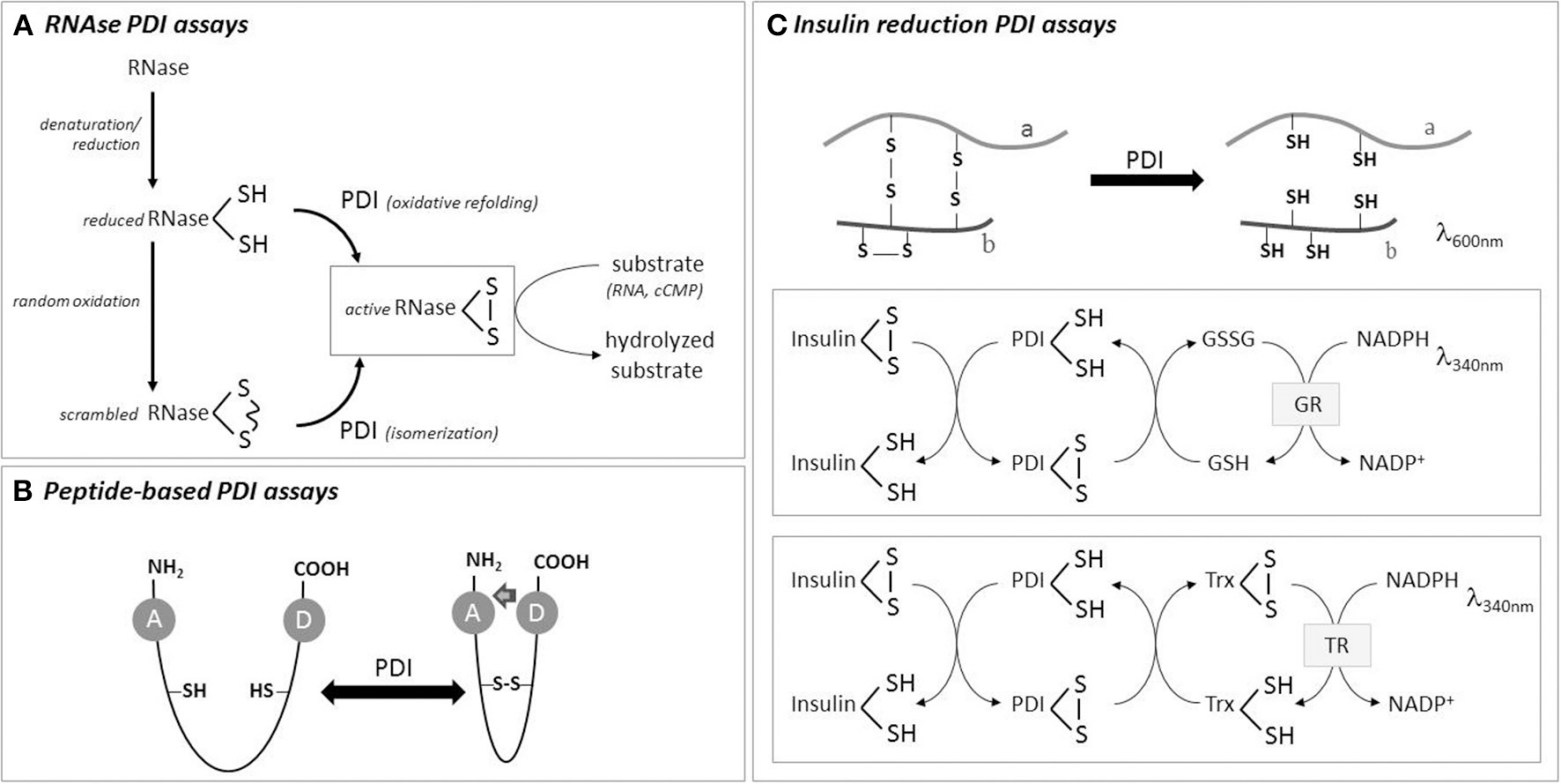
**Scheme of some PDI activity assays**. **(A)** RNase **with scrambled thiols** can be used as initial substrate for PDI isomerase assay, while totally reduced RNAse is used for PDI-mediated oxidative refolding assay. RNase gain-of-activity is measured by hydrolysis of its substrates, namely RNA or cyclic CMP. **(B)** Peptides can be used for oxidase, reductase or isomerase PDI assays, based on energy transfer from a donor to an acceptor residue with changes in fluorescence intensity. **(C)** PDI reduction assay is usually performed with insulin as substrate, which precipitates upon reduction of its B chain thiols. Coupling insulin reduction with NADPH consumption (using thioredoxin or glutaredoxin reductase systems) provides more precise quantitative results. See text for details.

For RNase, which is reduced in denaturing conditions and then allowed to oxidize under air at room temperature to acquire random disulfide bonds (Figure 1), aliquots are removed to measure substrate activity recovery during incubation with PDI. Partial folded RNAse can be quenched with excess alkylating agent before measuring RNAse activity recovery (Rancy and Thorpe, 2008), which is measured following the hydrolysis of 2 different RNAse substrates: high-molecular-weight RNA (Hillson et al., 1984) or cyclic cytidine monophosphate (cCMP) (El Hindy et al., 2014). The second provides higher increases in absorbance, but requires correction due to cCMP depletion over time and CMP-mediated RNase inhibition (Lyles and Gilbert, 1991). The assay buffer generally contains the redox pair GSH/GSSG, to drive PDI catalysis. One disadvantage of this method is that the most desirable is to obtain all experimental results from the same scRNase batch due to heterogeneity of scRNase among preparations. Otherwise, it is difficult to have reproducible results, independently if scRNase is home-made or commercial (Hatahet and Ruddock, 2009). This method has been extensively used in mechanistic PDI studies. It can also be applied in some particular cellular preparations, such as enriched-membrane fraction of vascular cells (Janiszewski et al., 2005) which does not contain soluble thioredoxin or glutaredoxin reductase systems, and thus may preferentially reflect PDI activity. Of note, it is important to confirm the presence of PDI in the analyzed specimen using western blotting (and even to use the amount of protein to normalize the activity).

The assay with RfBP is based on fluorescence quenching of free riboflavin due to its binding to apoRfBP (RfBP previously reduced under denaturing conditions to release riboflavin, followed by ferricyanide oxidation) (Rancy and Thorpe, 2008). ApoRfBP is mixed with riboflavin in the presence of PDI, and riboflavin fluorescence is measured directly. It is potentially a powerful assay, in which PDI is used in excess over substrate (30-fold), but is not a measurement of gain-of-function (Hatahet and Ruddock, 2009). This assay was not tested with biological samples yet. In homogenate samples the concentration of reagents would have to be optimized, in order to minimize the fluorescence interference of endogenous flavoenzymes, and secondarily, the interference of endogenous riboflavin binding proteins, such as riboflavin kinase or riboflavin transporters. This assay is not recommended for samples, such as plasma, with high levels of albumin or immunoglobulins (which also bind riboflavin).

### Oxidative refolding assay

Several fully reduced proteins can be used as substrates for PDI mediated-oxidative refolding. RNase and BPTI (3 disulfides) are considered the best model substrates of oxidative folding assay. In both cases, the intermediates formed during PDI-mediated oxidative folding are characterized by mass spectroscopy after thiol alkylation (Irvine et al., 2014). Although time-consuming, this experimental strategy is the best to estimate rate constants of PDI-dependent reactions during different steps of the folding process (Hatahet and Ruddock, 2009). Other frequently-used substrates include: lysozyme (4 disulfides, van den Berg et al., 1999) and 2 modified RNases, RNaseT1 (2 disulfides) and glutathionylated RNAseT1 (Ruoppolo and Freedman, 1995; Ruoppolo et al., 1996). Reduced RNaseT1 behaves in the assay as reduced RNase, while the glutathionylated substrate has to be deglutathionylated in the first step, a step that may also be mediated by PDI (Reinhardt et al., 2008; Townsend et al., 2009). Translation of oxidative refolding assays to biological samples has the same difficulties as those of isomerase activity discussed previously.

### Reduction assay

In this assay, PDI will reduce disulfides bonds present in the oxidized substrate. The most popular substrate, due to technical simplicity and low cost, is insulin. The reduction of insulin promotes the aggregation of insulin B chain and is followed by increase in turbidity. PDI is incubated with freshly prepared clear insulin solution in the presence of a reducing agent (DTT or GSH) (Holmgren, 1979). Better aggregation curves are obtained with B chain of insulin than A chain, which induces high background readouts (Karala and Ruddock, 2010). The kinetics of insulin reduction generates two parameters that can be used for relative quantification of different catalysts: the lag time (the time that takes to start precipitation, which can be set as an increase by 0.02 in absorbance over the baseline) and the rate of precipitation (the maximal absorbance increase per minute, Δabsorbance × min^−1^). It is the method elected for screening PDI inhibitors in diverse experimental setups (Khan et al., 2011; Paes et al., 2011; Jasuja et al., 2012), and can be optimized for HTPS (Smith et al., 2004) or coupled to fluorogenic dye in commercial kits (*ProteoStat™ PDI Assay Kit* from Enzo Life Sciences and *PDI Inhibitor Screening Assay Kit* from AbCam). It was also used in cellular homogenates. To increase assay sensitivity, fluorescent insulin (FITC-insulin) can be used. Also, in order to improve assay quantification, it is also possible to couple insulin reduction with NADPH consumption by coupling with GSSG (or thioredoxin reductase, Figure 1). In this case, one enzyme unit is defined as the amount of PDI that catalyzes the formation of GSSG per min, and kinetic parameters can be quantified (Vmax, Kobs, Ki). The assay has to be optimized in a way that insulin aggregation does not interfere with NADPH absorbance (e.g., 340 nm) (Morjana and Gilbert, 1991; Lee et al., 2014).

Other compound used for PDI reduction assay is GSSG. Although GSSG is a poor substrate for PDI (Hatahet and Ruddock, 2009), GSSG covalently attached to eosin (Di-E-GSSG) provides a good sensitivity to the assay, able to detect around nM PDI (Raturi and Mutus, 2007). Di-E-GSSG is non-fluorescent due to self-quenching of two proximal eosin moieties, but after reduction the probe shows ~70-fold increase in fluorescence. This method has been already used for biological samples, such as endothelial cell (HMEC-1) homogenates (Muller et al., 2013) and plasma (Prado et al., 2013). Finally, there is a radioactive method in which the release of radioactive I^125^-tyramine-SH is assessed, using I^125^-tyramine-SS-poly-(D-lysine) as substrate, bound to anionic cell surface. This method was employed in intact cells (myeloid and erythrocytes) to measure cell surface PDI reductase activity (Gallina et al., 2002), although confounding effects can potentially occur due to activity of proteases able to cleave the substrate (Xu et al., 2014).

### Peptides as PDI substrates

Some peptides have been developed to measure PDI activities *in vitro* and might have promising results when tested in biological samples. Peptides linked to fluorescent moieties allow high sensitivity and direct measurement of fluorescence. One example is a peptide based on tachyplesin I (TI), a 17-residue antimicrobial peptide that crosses membranes (Kersteen et al., 2005). Scrambled TI (4 cysteines) is isomerized by PDI to its native conformation in which a tryptophan and a dansyl-linked lysine physically interact, allowing fluorescence resonance energy transference (FRET). Another peptide, which can be used for reduction or oxidation PDI assays, depending on initial peptide redox state, contains two cysteines separated by a non-aethyleneglycol spacer (Christiansen et al., 2004). When reduced, this peptide is fluorescent due to o-aminobenzoyl group in the N-terminal region. Upon oxidation, o-aminobenzoyl fluorescence is quenched due to proximity with nitrotyrosine in the C-terminal segment. As a control, a peptide in which a cysteine is changed to serine can be used. Although they are not commercially available, both peptides show the advantages of enhanced homogeneity as starting materials (compared to scrambled thiol-proteins) and direct measurement of peptide fluorescence (i.e., aminobenzoyl/dansyl groups) within samples.

### Chaperone assay

Assays for chaperone-like activity of PDI are based on the catalysis by PDI of the self-refolding process of completely denatured substrates, which does not require disulfide bonds for folding. The first method for chaperone-like PDI activity was proposed to distinguish PDI chaperone from disulfide isomerase activity, using D-glyceraldehyde-3-phosphate dehydrogenase as substrate (Cai et al., 1994). Other substrates that can be used are lactate dehydrogenase, chemically denatured-rhodanese or citrate synthase, and thermally denatured-alcohol dehydrogenase (Xu et al., 2014). Briefly, the denatured protein is diluted in refolding buffer, in the presence of excess PDI (5–10-fold) and changes in aggregation are followed by light scattering or turbidity. Since changes in aggregation may not correlate with efficient substrate gain-of-function, many authors also measure the reactivation of the substrate, which requires the dissociation of PDI: substrate complex and optimization of substrate reactivation assay. One recently-reported assay uses green fluorescent protein as PDI substrate to increase sensitivity (Mares et al., 2011). However, it is important to note that oxidation can switch PDI conformation and interfere with its chaperone function (Wang et al., 2013).

## Critical comments on the measurement of PDI activity in biological samples

**Figure 2:**
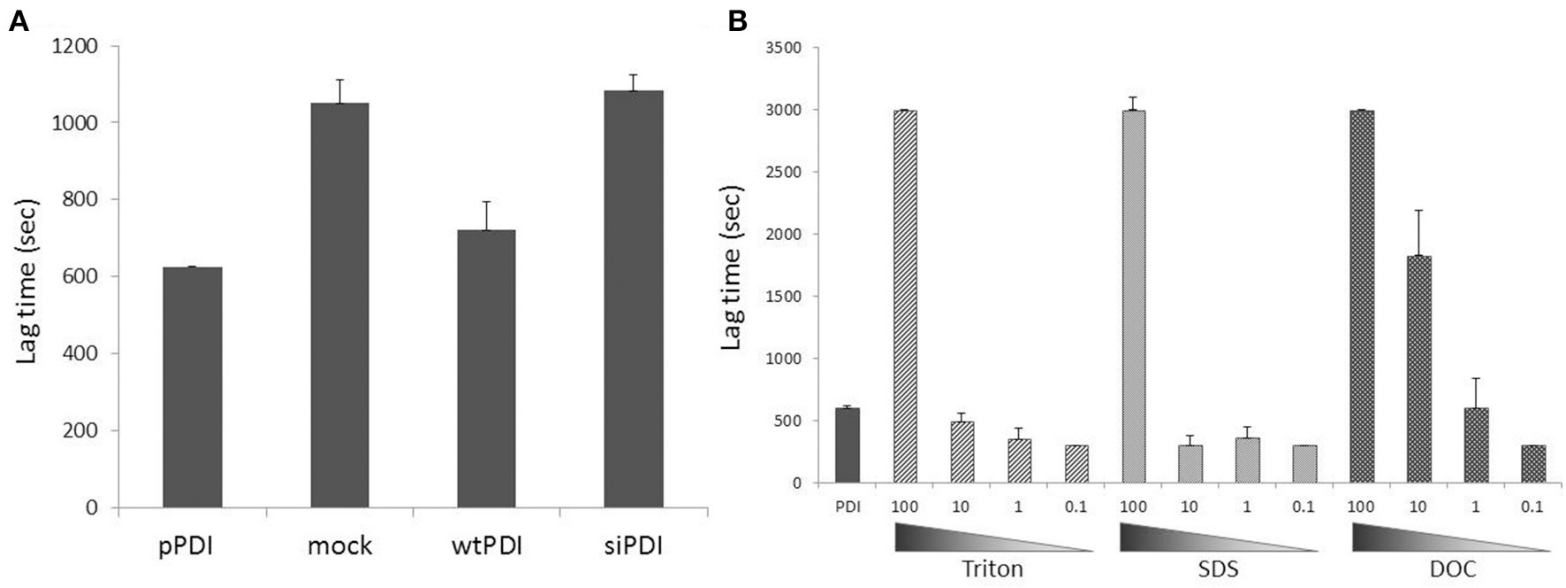
**Spurious inhibitors and detergents as PDI reductase assay interferents in biological samples. (A)** PDI overexpression (~3-fold increase vs. endogenous PDI) increased, while PDI silencing (by ca.70%) did not change insulin reduction in endothelial cell homogenates. Freshly prepared homogenates (150 μg) obtained by mechanical lysis (in the absence of detergents) were incubated with insulin (1 mg/mL) and PDI (1.5 μM) in the presence of DTT (1.5 mM) and insulin turbidity was assessed at 540nm. pPDI = purified PDI, mock = cells treated with transfection reagent, wtPDI = cells transfected with wild type PDI, siRNA = cells transfected with siRNA against PDI. Cells were lysed 24h after transfection procedure. Note the higher lag time for insulin reduction when exogenous PDI is incubated together with homogenates (mock), as compared with exogenous PDI alone (pPDI), suggesting that biological samples might have endogenous unknown PDI inhibitors. **(B)** Detergents commonly used in buffers in different experimental protocols can inhibit or increase insulin reduction mediated by PDI in vitro, depending on their concentration. PDI (1.5 μM) was incubated with insulin at the same experimental conditions of **(A)**. Detergents (nonionic Triton X-100, anionic sodium dodecyl sulfate or anionic sodium deoxycholate) were added at concentrations 100-fold (150 μM) to 0.1-fold (0.15 μM) vs. PDI concentration. Lag time was calculated as time for absorbance reaching 0.1 unit.

All the assays discussed above can be, in theory, applied to biological samples since in general the latter contain high amounts of PDI. With the combination of specific PDI inhibitors, one may guarantee that the activity measured in the whole homogenates is in fact due to PDI and not to other reductase or reduction systems. However, in practice it not as simple as that. Indeed, so far only reductase assays were reportedly transposed to cellular samples. Measurement of reductase PDI activity in cell surface is less prone to endogenous interferents, and the assay can be combined with a neutralizing PDI antibody (Raturi and Mutus, 2007; Langer et al., 2013). In whole cell homogenates, reductase assays will be affected by other reductase systems such as thioredoxin or glutaredoxin. Indeed, the fluorescent Di-E-GSSG was recently employed to measure thioredoxin reductase activity in serum, plasma and lymphocyte lysate (Montano et al., 2014). Furthermore, biological samples might have endogenous unknown PDI inhibitors, as suggested by the observations from our laboratory indicating higher lag time for insulin reduction when exogenous PDI is incubated together with homogenates, as compared to exogenous PDI alone (Figure 2A). These observations reinforce the use of a panel of PDI inhibitors and/or the strategy of PDI gain/loss-of-function to provide reliable results for PDI activity assays. For example, in endothelial cells PDI reductase activity was increased by 2-fold after overexpression of wild-type PDI, while no such increase was detected in cells overexpressing PDI mutated in all redox thiols (Muller et al., 2013). Using the coupled insulin reduction assay, Lee et al. (2014) showed that overexpression of both PDI and thioredoxin interaction protein (Txnip) increased NADPH consumption in cell homogenates compared to PDI transfected controls. In vascular cells, PDI overexpression decreased the lag time of insulin reduction kinetics, although PDI silencing had no effect (Figure 2A). These data also suggest that in complex samples, results obtained with PDI reduction assays might significantly reflect PDI expression levels rather than “intrinsic” PDI isomerase activity within cell. One possible alternative is to measure PDI activity after its immunoprecipitation, which reduces contaminants from the whole homogenate.

Several molecules are known to bind and inhibit PDI activity *in vitro* (e.g., nitric oxide, the hormones T3 and estrogen, the peptides somastotin and bacitracin, synthetic organic compounds as bisphenol A and phenylarsenic oxide). However, only few are able to inhibit PDI in some particular experimental *in vivo* conditions. Examples are the reversible PDI inhibitors derived from plants, juniferdin (Khan et al., 2011) and quercetin-3-rutinoside (Jasuja et al., 2012), as well as synthetic compounds that irreversibly inhibit PDI, 16F16, thiomuscimol (Hoffstrom et al., 2010), RB-11-ca (Banerjee et al., 2013), and PACMA31(Xu et al., 2012). These compounds may be used as additional controls during PDI activity assays, although none of these compounds to date have been shown to specifically inhibit PDI. The neutralizing anti-PDI (RL90) antibody is also a powerful control through its specificity, although recently Wu et al. (2012) showed some cross-reactivity with another member of PDI family, Erp57, in Di-E-SSG assay.

Another important experimental topic is the buffer used for cell and tissue lysis. In general they contain, in addition to reducing agents, one or more surfactants, necessary for cell membrane rupture. For example, the widely used RIPA lysis buffer contains NP-40 (1%, 16 mM), sodium deoxycholate (0.5%, 12 mM) and sodium dodecyl sulfate (0.1%, 3.5 mM) (Cold Spring Harbor Protocols). However, Triton was already shown to bind PDI b' domain and strongly inhibit enzyme activity (Klappa et al., 1998), and is typically used at 0.05% (0.77 mM) to inhibit purified PDI in reduction assay (Karala and Ruddock, 2010). Indeed, results from our laboratory indicate that surfactants with distinct physicochemical properties significantly alter PDI reductase activity *in vitro* (Figure 2B, same for NP40, CHAPS and saponin). Inhibition or even a small increase of PDI-mediated insulin reduction depends on the proportion between PDI and detergent molecules (Figure 2B). Thus, the best procedure for PDI activity assays is to obtain protein extracts by mechanical lysis in detergent-free buffers.

## Conclusions and perspectives

Understanding the role of PDI in cells and tissues as a homeostatic redox signaling adaptor with chaperone properties will require different assays to assess PDI activity. Currently, a major challenge is to precisely measure overall PDI isomerase activity in cells and tissues, and not only one step of thiol rearrangement (oxidation or reduction). In addition, the assay should be performed preferentially *in situ*, since PDI activity may change depending on cell location or redox status. As long as the ideal PDI isomerase assay is yet unavailable for cells and tissues, PDI reductase activity has been measured instead in the cell surface and intact cells, as well as cell homogenates. Such results should be interpreted with care. Improvements in assay reliability will probably require not only advances into sensitivity and specificity, but optimization of adequate controls as well (neutralizing PDI antibodies or more specific emerging PDI inhibitors), or even cell fractionation to remove other reductase systems. Optimization of assays with fluorescent peptides for biological samples seems to be a forthcoming promising approach.

### Conflict of interest statement

The authors declare that the research was conducted in the absence of any commercial or financial relationships that could be construed as a potential conflict of interest.
